# Non-invasive urinary metabolomic profiling discriminates prostate cancer from benign prostatic hyperplasia

**DOI:** 10.1007/s11306-017-1194-y

**Published:** 2017-03-09

**Authors:** Clara Pérez-Rambla, Leonor Puchades-Carrasco, María García-Flores, José Rubio-Briones, José Antonio López-Guerrero, Antonio Pineda-Lucena

**Affiliations:** 1 Structural Biochemistry Laboratory, Centro de Investigación Príncipe Felipe, 46012 Valencia, Spain; 2 Laboratory of Molecular Biology, Fundación Instituto Valenciano de Oncología, 46009 Valencia, Spain; 3 Department of Urology, Fundación Instituto Valenciano de Oncología, 46009 Valencia, Spain; 4 Drug Discovery Unit, Instituto de Investigación Sanitaria La Fe, Avda. Fernando Abril Martorell, 106, 46026 Valencia, Spain

**Keywords:** Biomarkers, Metabolomics, Prostate cancer, Benign prostatic hyperplasia, Nuclear magnetic resonance

## Abstract

**Introduction:**

Prostate cancer (PCa) is one of the most common malignancies in men worldwide. Serum prostate specific antigen (PSA) level has been extensively used as a biomarker to detect PCa. However, PSA is not cancer-specific and various non-malignant conditions, including benign prostatic hyperplasia (BPH), can cause a rise in PSA blood levels, thus leading to many false positive results.

**Objectives:**

In this study, we evaluated the potential of urinary metabolomic profiling for discriminating PCa from BPH.

**Methods:**

Urine samples from 64 PCa patients and 51 individuals diagnosed with BPH were analysed using ^1^H nuclear magnetic resonance (^1^H-NMR). Comparative analysis of urinary metabolomic profiles was carried out using multivariate and univariate statistical approaches.

**Results:**

The urine metabolomic profile of PCa patients is characterised by increased concentrations of branched-chain amino acids (BCAA), glutamate and pseudouridine, and decreased concentrations of glycine, dimethylglycine, fumarate and 4-imidazole-acetate compared with individuals diagnosed with BPH.

**Conclusion:**

PCa patients have a specific urinary metabolomic profile. The results of our study underscore the clinical potential of metabolomic profiling to uncover metabolic changes that could be useful to discriminate PCa from BPH in a clinical context.

## Introduction

Prostate cancer (PCa) is the most common cancer in men worldwide. The number of PCa cases is increasing, nowadays representing the sixth leading cause of cancer deaths in men (Zhang et al. 2014). Currently, the most frequently used tests for PCa screening include the determination of prostate specific antigen (PSA) serum levels and digital rectal examination (DRE) (Bunting 2002). The introduction of PSA testing revolutionised PCa screening and became widely adopted by the early 1990s. Since then, the European Randomized study of Screening for Prostate Cancer (ERSPC) has reported a small absolute survival benefit with PSA screening (Ilic et al. 2013; Heijnsdijk et al. 2015). However, PCa screening suffers from a number of limitations, due to the poor specificity of PSA test for detecting cancer and for differentiating indolent cancers from high risk ones.

The low specificity of serum PSA has translated into many unnecessary prostate biopsies and overtreatment of tumours with a low malignant potential, or with a low potential for morbidity or death if left untreated (Draisma et al. 2003; Zappa et al. 1998). It has been estimated that the overdiagnosis, and consequently the overtreatment, of PCa ranges between 30 and 84%, depending on the studies (Etzioni et al. 2002; McGregor et al. 1998). Moreover, trans-rectal ultrasound (TRUS)-guided biopsy following histopathology-based Gleason score, the gold standard test providing histological confirmation (Gleason 1977), is also plagued by high false negative rates (Rabbani et al. 1998; Schoenfield et al. 2007). Early-stage PCa is generally not visible on ultrasound, thus meaning that many tumours are missed on initial biopsy and patients are required to undergo repeated prostate biopsies before definitive PCa detection.

Very few biomarkers are currently validated for use in PCa diagnosis. A recent FDA clinical-grade urine-based assay for the non-coding transcript *PCA3* (overexpressed in >95% of PCa) has demonstrated utility when combined with serum PSA for PCa detection (Loeb and Partin 2011). Another potential biomarker is the specific TMPRSS2 and ERG rearrangement at 21q22, which is 100% indicative of PCa (Barbieri et al. 2012). However, it is only present in approximately 50% of PCa cases. Hence, additional clinically robust biomarkers able to differentiate between indolent and aggressive PCa are urgently needed.

In this context, metabolomics could represent an alternative and very powerful approach for the understanding of the biological pathways and molecular mechanisms involved in the onset and progression of PCa. Metabolomics focuses on the characterisation of metabolic signatures in biofluids or tissues and is leading to advanced diagnostic and therapeutic procedures (Nicholson et al. 2005). Recent studies have shown the potential of metabolomic approaches in the PCa field (Kumar et al. 2015; Stabler et al. 2011; Struck-Lewicka et al. 2015; Zhang et al. 2013). However, so far, no comprehensive PCa studies have been performed on urine, the most accessible and least invasive biofluid, using Nuclear Magnetic Resonance (^1^H-NMR) spectroscopy, a robust and reliable technological platform allowing the simultaneous measurement and quantification of metabolites with minimal sample handling (Duarte and Gil 2012).

To that end, in this study, a thorough analysis of the urinary metabolomic profile of PCa patients was compared with that corresponding to individuals diagnosed with benign prostatic hyperplasia (BPH), a prostatic condition that cannot be easily distinguished from PCa based on the current PSA screening (Roehrborn et al. 1999). Using a metabolomic approach based on ^1^H-NMR, it was possible to identify a set of specific metabolites that could contribute to a better understanding of the pathophysiological processes involved in the onset and progression of this disease.

## Materials and methods

### Patient selection

Patient recruitment was carried out through the Department of Urology and the Biobank of the Instituto Valenciano de Oncología (Valencia, Spain), and measurement and analysis of the urinary metabolomic profiles were performed at the Centro de Investigación Príncipe Felipe (Valencia, Spain) and the Instituto de Investigación Sanitaria La Fe (Valencia, Spain). Urine samples were collected from 64 PCa patients and 51 age-matched individuals. Patient recruitment and sampling procedures were performed in accordance with the Declaration of Helsinki and applicable local regulatory requirements and laws and after approval from the Ethics Committee of the Instituto Valenciano de Oncología. Written informed consent was obtained from each participant before being included in this study.

Clinical diagnosis of individuals was performed according to serum PSA, DRE, biopsy results and Gleason score. Biopsy was performed using at least 6 cores and classification of the individuals included in the study was carried out according to the EAU-ESTRO-SIOG Guidelines on Prostate Cancer (Mottet et al. 2016). The control group consisted of men with no proven PCa based on PSA levels, negative findings on DRE and no malignant findings in prostate tissue biopsies. Based on their clinical characteristics, all of them were diagnosed with BPH. Clinical and demographics characteristics of the individuals included in the study are shown in Table 1.

**Table 1:** Characteristics of the individuals included in the study

	BPH group (*n* = 51) (median, range)	PCa patients (*n* = 64) (median, range)
Age (years)	62.1 (41.4–74.5)	66.2 (50.0–86.3)
BMI (kg m^−2^)	27 (22.8–34)	27.5 (23–33)
Prostate volume (ml)	52 (24–171)	40.5 (2–134)
PSA (ng/mL)	4.86 (1.02–11.29)	5.11 (0.85–71.41)
Number of cores	12 (10–19)	12.5 (6–54)
Positive cores	NA	25% (0.05–100)
Tumor burden	NA	3.71% (0.14–67.74)
Tumor gleason score	NA	6 (5–9)

*BPH* benign prostatic hyperplasia, *PCa* prostate cancer, *PSA* prostate-specific antigen, *BMI* body mass index, *NA* not applicable

### Sample preparation and ^1^H-NMR acquisition

Urine samples were immediately frozen after collection and stored at −80 °C. At the time of ^1^H-NMR analysis, urine samples were thawed on ice and centrifuged at 6000 rpm for 5 min at room temperature. 60 µL of 1.5 mol/L potassium phosphate buffer (pH 7.4) containing 0.1% trimethylsilylpropionic acid-d_4_ sodium salt (TSP) and 0.05% NaN_3_ were added to 540 µL of urine sample supernatant. After this, 500 µL of the mixture were transferred to a 5-mm NMR tube for analysis.

^1^H-NMR spectra were acquired using a Bruker Avance II 500 MHz spectrometer. ^1^H-NMR experiments were acquired at 310 K for every sample. Carr-Purcell-Meiboom-Gill (CPMG) spin-echo pulse sequence (Meiboom and Gill 1958), which generates spectra edited by T2 relaxation times with reduced signals from high molecular weight species and giving improved resolution of low molecular weight metabolite resonances, was collected for each sample with a total of 16 accumulations and 72 K data points over a spectral width of 16 ppm. A 4-s relaxation delay was included between free induction decays (FIDs). The total spin–spin relaxation delay was 40 ms. A one-dimensional (1D) NOESY pulse sequence that generates an unedited spectrum with improved solvent peak suppression (Nicholson et al. 1995) was collected using the same parameters as the CPMG experiment, with a 4-s relaxation delay and 10 ms of mixing time. For both experiments, a water presaturation pulse of 25 Hz was applied throughout the relaxation delays to improve solvent suppression. In addition, two-dimensional (2D) J-resolved spectra, homonuclear 2D ^1^H–^1^H total correlation spectroscopy and 2D ^1^H, ^13^C heteronuclear single quantum correlation were acquired for selected samples to facilitate the identification of biochmemical substances (Beckonert et al. 2007). All spectra were multiplied by a line-broadening factor of 1 Hz and Fourier transformed. Spectra were automatically phased and baseline corrected, and chemical shift internally referenced to the methyl group signal of TSP at 0.00 ppm using TOPSPIN 3.0 (Bruker Biospin).

### Data modelling and statistical analysis

**Fig. 1:**
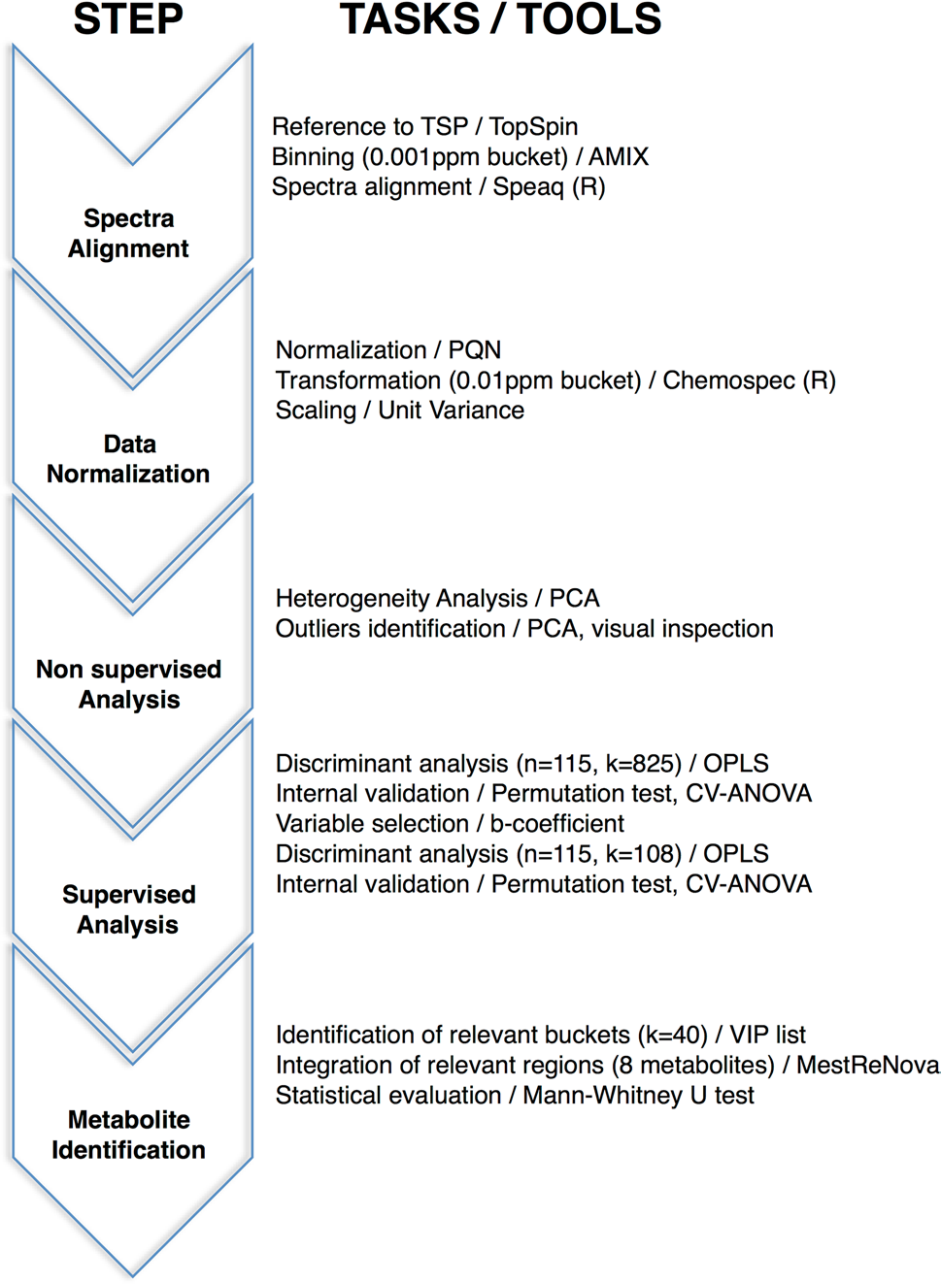
General scheme of the data modeling and statistical analysis procedures with the main steps highlighted (*n* number of samples, *k* number of variables)

The main steps of the data modelling and statistical analysis procedures followed in this study are shown in Fig. 1. 1D CPMG spectra were binned using Amix 3.9.7 (Bruker Biospin) into 0.001 ppm wide rectangular buckets over the region δ 9.50–0.15 ppm. The water (δ 5.09–4.55 ppm) and urea signal (δ 6.10–5.52 ppm) regions were excluded from the analysis to avoid interferences arising from differences in water suppression and variability from urea signal, respectively. Spectra were aligned using the “Speaq” R package, a hierarchical cluster-based peak alignment algorithm that minimizes chemical shift variations (Vu et al. 2011), and normalization of the aligned spectra was performed according to the probabilistic quotient normalization method (PQN) (Dieterle et al. 2006). Finally, the resulting bucket table was transformed into a data matrix containing 0.01 ppm wide rectangular buckets using the “Chemospec” R package (Hanson 2014) to facilitate the statistical analysis.

Multivariate statistical analysis was carried out using SIMCA-P 12.0 (Umetrics AB). Before statistical analysis, data were scaled to unit variance by dividing each variable by 1/SD, where SD represents the standard deviation value of each variable, so that all variables were given equal weight regardless of their absolute value. Principal component analysis (PCA), a nonsupervised statistical approach, was performed on normalized data for finding potential patterns, intrinsic clusters, and outliers. Orthogonal partial least squares discriminant analysis (OPLS-DA) was applied to minimize the possible contribution of inter-group variability and to further improve separation between the groups of samples. The default method of sevenfold internal cross validation was applied, from which Q^2^Y (predictive ability parameter, estimated by cross-validation) and R^2^Y (goodness of fit parameter) values were extracted. Those parameters, together with the corresponding permutation tests (*n* = 100), were used for the evaluation of the quality of the OPLS-DA models obtained. Variable selection was based on the regression coefficient (b-coefficient) method (Diaz et al. 2013), retaining only those variables with a quotient |b/b_cvSE_| > 1.0, being b_cvSE_ the standard error associated with the b-coefficients.

### Identification and quantification of relevant metabolites

The identification of the variables responsible for the separation between groups of samples in the OPLS-DA models was performed according to the corresponding loading plots and the variable importance in projection (VIP) list of each model. Metabolites of interest were identified using Analysis of MIXtures (AMIX; Bruker) in combination with the Bruker NMR Metabolic Profiling Database BBIOREFCODE 2.0.0 database (Bruker Biospin, Rheinstetten, Germany), as well as other existing public databases and literature reports (Bouatra et al. 2013; Salek et al. 2007). Metabolites contributing to group discrimination in each model were integrated using MestReNova (Cobas and Sardina 2003) to enable comparison between sample groups. Statistical significance of the observed changes was assessed using the Mann–Whitney U test. A *p* value lower than 0.05 (confidence level 95%) was considered statistically significant.

## Results

### Urinary metabolomic profile of PCa patients

**Fig. 2:**
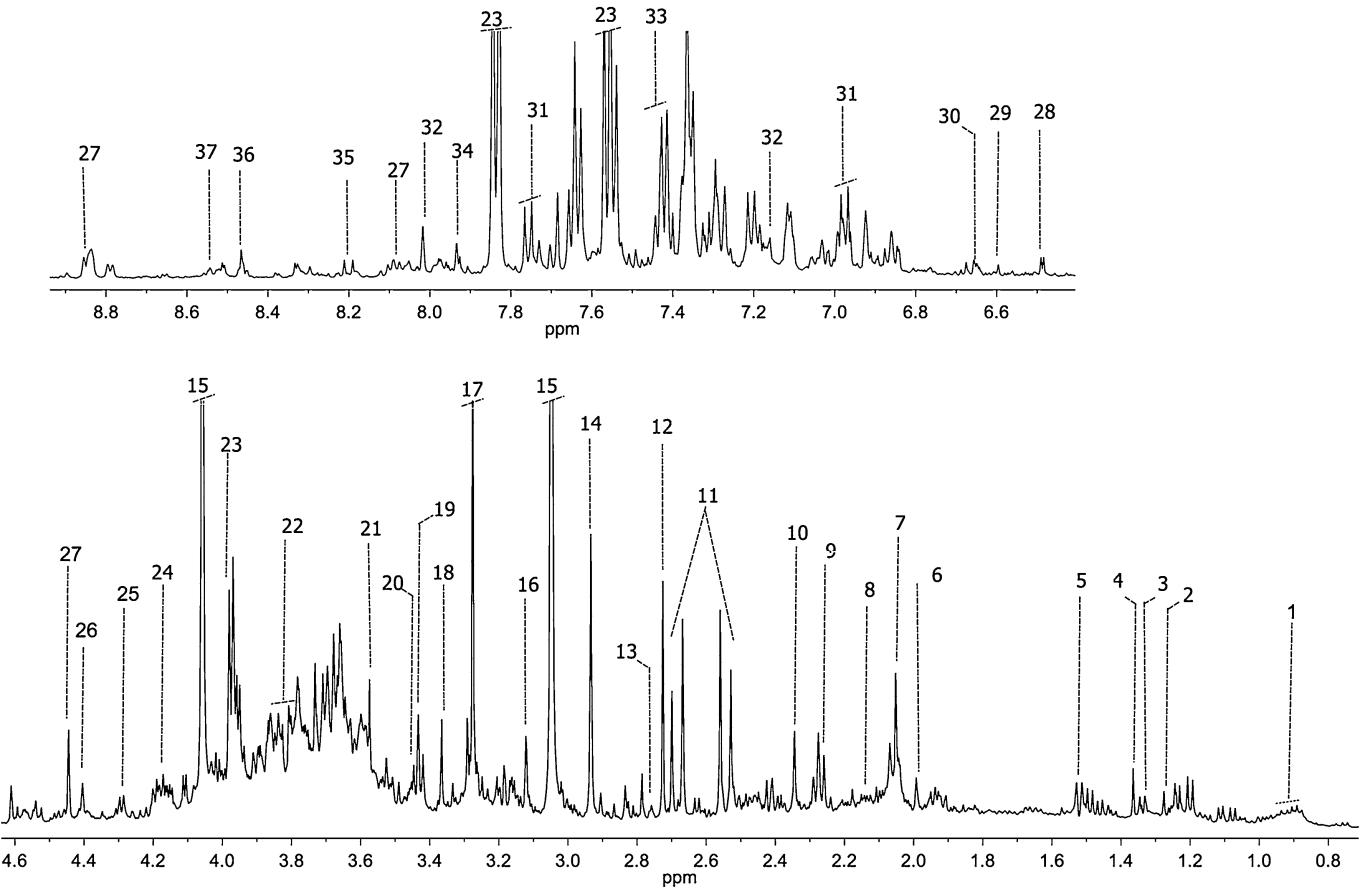
Representative 500 MHz ^1^H-NMR spectrum and assignment of a urine sample from a PCa patient. Assigned metabolites:* 1* branched-chain amino acids;* 2* 3-hydroxyisovalerate;* 3* lactate;* 4* 2-hydroxyisobutyrate;* 5* alanine;* 6* acetate; *7* *N*-acetyl groups;* 8* glutamate;* 9* 2-hydroxy-glutarate;* 10* pyruvate;* 11* citrate;* 12* dimethylamine;* 13* sarcosine;* 14* dimethylglycine;* 15* creatinine;* 16* cis-aconitic acid;* 17* trimethylamine-*N*-oxide;* 18* methanol;* 19* trans-aconitic acid;* 20* taurine;* 21* glycine;* 22* serine;* 23* hippurate;* 24* pseudouridine;* 25* threonine;* 26* dihydroxyacetone;* 27* trigonelline;* 28* U1;* 29* fumarate;* 30* 2-furoylglycine;* 31* 4-hydroxybenzoate;* 32* 3-methylhistidine;* 33* phenylalanine;* 34* histidine;* 35* hypoxanthine;* 36* formate;* 37* 4-imidazole-acetate

^1^H-NMR CPMG spectra were acquired for all urine samples included in the study. Good quality spectra, characterized by the presence of signals with varying degrees of overlapping, were obtained for most of the samples. Figure 2 displays a representative urine ^1^H-NMR spectrum from a PCa patient and the assignment of the most relevant metabolites identified in these samples. In general, spectra corresponding to this biofluid contain signals from a wide range of low-molecular-weight metabolites of diverse chemical classes (Bouatra et al. 2013), including organic acids, simple sugars and polysaccharides, amino acids, and low-molecular-weight proteins. In particular, urine spectra are dominated by urea, creatinine, trimethylamine-*N*-oxide, dimethylamine, hippuric acid, and citric acid resonances, among others (Fig. 2).

### Non-supervised analysis of the urinary metabolomic profiles

Sample homogeneity within the groups of samples was based on the PCA analysis of the ^1^H-NMR CPMG urine spectra. Using this approach, it was possible to identify urine samples exhibiting metabolic profiles unusually different to the rest of the samples within their groups. Careful inspection of those samples revealed their spectra contained signals corresponding to several contaminants (e.g., manitol, ethanol, drugs, etc.), or exhibited bad quality due to acquisition problems. These samples were classified as outliers and excluded from the study.

PCA analysis was also used to evaluate the potential influence of different clinical variables on the metabolic profiles obtained for the urine samples of PCa patients and individuals diagnosed with BPH. None of the variables assessed (i.e., age, PSA level, body mass index (BMI), Gleason score) had an impact in the clustering of the samples from both groups. Finally, a non-supervised analysis of the global data did not reveal any significant clustering of the samples based on the urine metabolomic profiles of the two sample groups in this study.

### Supervised analysis of the urinary metabolomic profiles

**Fig. 3:**
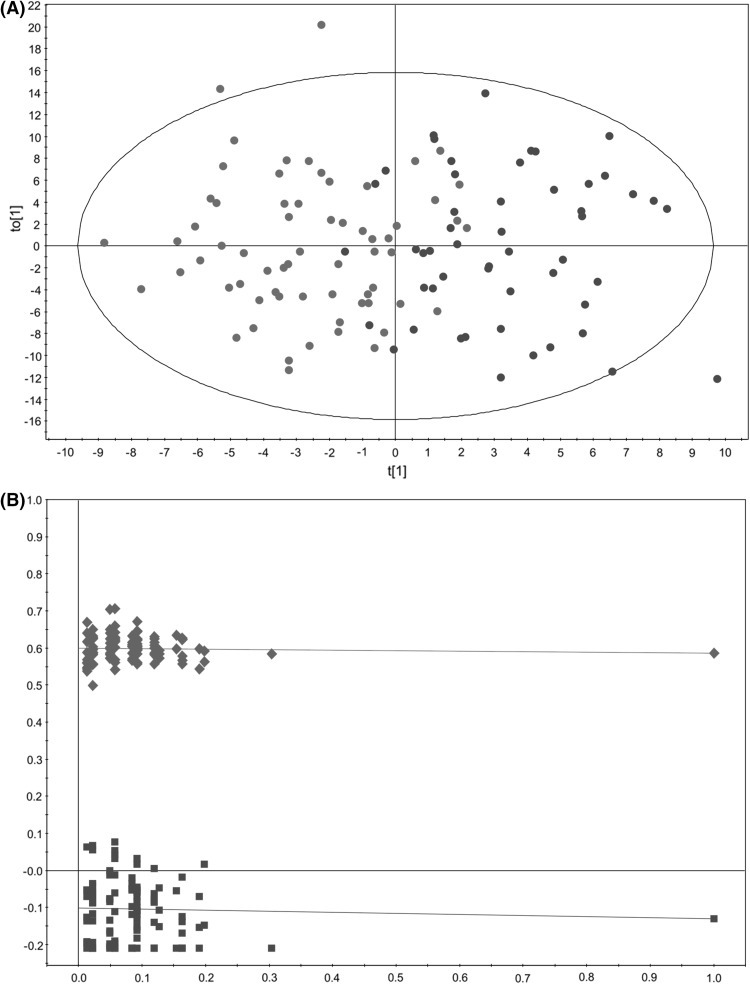
Multivariate modelling resulting from the analysis of urine ^1^H-NMR spectra before variable selection (823 variables). **a** OPLS-DA score plot for the comparison between PCa patients (*red circle*) *vs*. individuals diagnosed with BPH (*blue circle*); **b** internal validation of the corresponding OPLS-DA model by permutation analysis (n = 100), R^2^ (*green diamond*), Q^2^ (*blue square*)

To better examine potential differences between the groups of samples, an OPLS-DA model aiming to discriminate the urinary profiles from PCa patients and individuals diagnosed with BPH was built. This OPLS-DA model (Fig. 3) showed a reasonable fitting of the data (R^2^ = 0.586), but it did not exhibit any predictive power (Q^2^ = −0.230). OPLS-DA model significance was assessed using a cross-validated ANOVA (*p* ≤ 0.01 was considered significant) and a permutation test (*n* = 100). The results of this internal validation (R^2^ = 0.600, Q^2^ = −0.101; *p* value >0.01) revealed overfitting of the data, most probably reflecting the elevated number of variables (823) over samples (115) used to build this model (Andersen and Bro 2010).

**Fig. 4:**
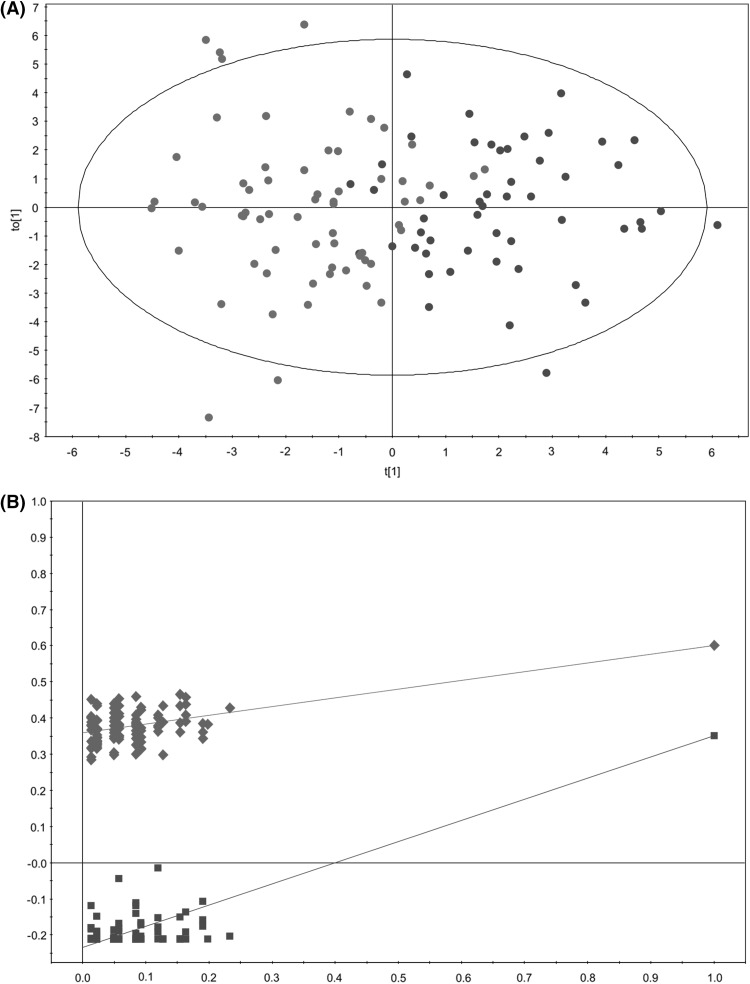
Multivariate modelling resulting from the analysis of urine ^1^H-NMR spectra after variable selection (108 variables). **a** OPLS-DA score plot for the comparison between PCa patients (*red circle*) vs. individuals diagnosed with BPH (*blue circle*); **b** internal validation of the corresponding OPLS-DA model by permutation analysis (n = 100), R^2^ (*green diamond*), Q^2^ (*blue square*)

To overcome this limitation, a variable selection strategy, based on the regression coefficient method (b-coefficient) (Diaz et al. 2013), was followed to remove uninformative variables. The application of this variable selection method reduced the number of variables to 108, and the OPLS-DA then provided a model with significant reduction in sample scores dispersion and improved predictive power (Q^2^ = 0.416) (Fig. 4). The results of the internal validation of this new OPLS-DA model (R^2^ = 0.358, Q^2^ = −0.234; *p* value <0.01) confirmed its robustness (Szymanska et al. 2012). The value of R^2^ of this new model remained unchanged (R^2^ = 0.600) when compared with the original one, confirming that the discarded variables were not relevant for explaining the differences between the metabolomic profiles of PCa patients and individuals diagnosed with BPH.

### Metabolite identification and quantification

Examination of the corresponding loading plot and VIP list of the new OPLS-DA model facilitated the identification of the most relevant variables that were contributing to the discrimination of the PCa patients and the individuals diagnosed with BPH. Following this strategy, a total of 40 out of the 108 variables were identified as relevant regions in the discrimination, and used to identify the spectral signals corresponding to the altered metabolites in pathological conditions. The metabolites corresponding to those regions were identified through a combination of their ^1^H chemical shifts in the ^1^H-NMR CPMG spectra and the spin system patterns obtained from the 2D spectra acquired for representative samples of each group.

Further analysis of the data was carried out with the use of variable-size bucketing to assess if the metabolites associated with the relevant variables were also significant when comparing the two sample groups. This analysis revealed a total of 8 metabolites (Table 2) whose concentrations exhibited statistically significant differences when comparing the urinary metabolomic profiles of PCa patients and individuals diagnosed with BPH. Thus, it was found that urine from PCa patients, compared with individuals diagnosed with BPH, was characterized by increased concentrations of branched-chain amino acids (BCAA), glutamate and pseudouridine, and decreased concentrations of glycine, dimethylglycine, fumarate, 4-imidazole-acetate, and one unknown metabolite (U1).

**Table 2:** Mean intensities and variations for the statistically significant metabolites involved in the discrimination between individuals diagnosed with BPH and PCa patients

Metabolite	δ ^1^H (ppm)^a^	BPH group (mean ± s.e.m.)^b^	PCA patients (mean ± s.e.m.)^b^	*p* value	% variation
BCAA	0.930–0.842	13.10 ± 0.26	14.33 ± 0.37	0.011*	9.37
Glutamate	2.115–2.081	11.72 ± 0.18	12.42 ± 0.21	0.012*	5.98
Dimethylglycine	2.944–2.922	20.56 ± 0.91	17.17 ± 1.11	0.034*	−16.48
Glycine	3.582–3.567	23.29 ± 1.01	20.49 ± 0.94	0.015*	−12.00
Pseudouridine	4.309–4.277	6.88 ± 0.13	7.68 ± 0.41	0.049*	11.65
U1	6.496–6.478	0.69 ± 0.09	0.45 ± 0.04	0.027*	−34.50
Fumarate	6.551–6.498	0.99 ± 0.05	0.87 ± 0.03	0.021*	−12.54
4-Imidazole-acetate	8.567–8.517	1.77 ± 0.11	1.37 ± 0.62	0.006*	−22.52

*BCAA* branched-chain amino acids, *ppm* parts per million, *s.e.m*. standard error of mean. *P* values calculated using the Mann–Whitney U test. *P < 0.05

^a^Chemical shift range for integration

^b^Spectral intensity in arbitrary units

## Discussion

Efforts to identify non-invasive PCa biomarkers that can stratify patients with high sensitivity and specificity for screening, diagnosis, prognosis, prediction and monitoring remain a fundamental goal in this area (Thapar and Titus 2014). In this context, our study represents the first comprehensive study focused on the characterisation and comparison of the specific urinary metabolomic profile of PCa patients with that of patients diagnosed with BPH using ^1^H-NMR. The only other report focused on the analysis of a relatively similar set of urine samples to that included in our study suggested that “fingerprints” (i.e., global profiles) based on the analysis of urinary NMR metabolomic profiles could be a suitable and promising method for PCa detection (Zaragoza et al. 2014). The in-depth analysis carried out in our study, based on non-invasive urinary metabolomic studies, reveals that the discrimination between PCa patients and individuals diagnosed with BPH actually relies on specific urinary metabolites, an information that could eventually contribute to the early diagnosis of PCa. Our results show that the urinary metabolomic profile of PCa patients, compared with individuals diagnosed with BPH, is characterised by statistically significant changes in the concentration of several metabolites. The analysis of those metabolic alterations reveals that PCa is associated with profound changes in energy metabolism.

Thus, in our study, we observed decreased levels of glycine and dimethylglycine when the urinary metabolomic profiles of PCa patients and individuals diagnosed with BPH were compared. This result is in agreement with recent studies performed in serum (Kumar et al. 2015) and urine (Struck-Lewicka et al. 2015) of PCa patients and healthy individuals. Kumar et al. (Kumar et al. 2015) found increased levels of sarcosine and decreased levels of glycine in serum samples of PCa patients compared with healthy individuals. Furthermore, Struck-Lewicka et al. (Struck-Lewicka et al. 2015) have reported decreased levels of glycine, in a study performed by liquid chromatography–mass spectrometry (LC–MS) and gas chromatography–mass spectrometry (GC–MS), when comparing the urinary metabolomic profiles of PCa patients and healthy individuals.

Glycine is converted to sarcosine, an *N*-methyl derivative of glycine that has been previously linked to PCa (Sreekumar et al. 2009), by the enzyme glycine-*N*-methyltransferase (GNMT). Sarcosine levels are also regulated by sarcosine dehydrogenase (SARDH), the enzyme that converts sarcosine back to glycine, and dimethylglycine dehydrogenase (DMGDH) which generates sarcosine from dimethylglycine (Sreekumar et al. 2009). The involvement of sarcosine in PCa has been the subject of many studies (Khan et al. 2013; Miyake et al. 2012; Issaq 2011; Bianchi et al. 2011; Lucarelli et al. 2012; Sreekumar et al. 2009; Kumar et al. 2015; Jentzmik et al. 2010). However, its role as a potential biomarker of PCa remains controversial and unclear (Ploussard and de la Taille 2010). In our study, we found elevated levels of sarcosine in PCa patients, although this variation was not statistically significant. Taken together, our results would support the idea of an interconversion between glycine/dimethylglycine and sarcosine through the activation of both DMGH and GNMT, and the down-regulation of SARDH.

There are also other mechanisms that could contribute to a reduction in the levels of circulating glycine. Recent work on cancer metabolomics has shown that glycine uptake is associated with cancel cell proliferation through its involvement in one-carbon metabolism (Zhang et al. 2012). This pathway has been traditionally considered a “housekeeping” process, and encompasses a complex metabolic network based on the chemical reactions of folate compounds. Recent findings also suggests that hyperactivation of this pathway could potentially be a driver of oncogenesis and tumor maintenance (Locasale 2013). In this context, glycine metabolism has been reported to be involved in cell transformation and tumorigenesis. This process would be mediated by the activity of glycine dehydrogenase (decarboxylating) (GLDC) that links glycine cleavage with the charging of the folate cycle.

Furthermore, the rapid, dysregulated cell growth found in cancer cells, demands extra sources of energy to sustain proliferation (Zhang et al. 2012). Thus, in addition to pyruvate derived from glycolysis, fatty acids and particularly amino acids can supply substrates to the tricarboxylic acid (TCA) cycle to maintain mitochondrial production in cancer cells (Chen and Russo 2012).

One of the factors contributing to the availability of amino acids is a metabolic syndrome experienced by approximately 60% of PCa patients termed cachexia (Utech et al. 2012). This process involves a net increase in protein catabolism along with activation of proteolysis, and has a tremendous impact in the levels of BCAAs (O’Connell 2013). Under normal conditions, BCAA oxidation in skeletal muscle provides 6–7% of the energy needs, but under highly catabolic circumstances, such as cancer cachexia, the contribution can be as high as 20% (Lam and Poon 2008). In these conditions, it would be expected an increase in circulating BCAAs, thus being in perfect agreement with our observation and other studies carried out in prostate tissue (Giskeødegård et al. 2013; McDunn et al. 2013) and serum samples (Giskeødegård et al. 2015) from PCa patients. It would also explain the results obtained in previous studies showing that the levels of BCAAs are significantly increased in certain neoplastic processes (e.g., gastric and esophageal cancers) (Fan et al. 2012; Zhang et al. 2013). Interestingly, BCAAs can be converted into acetyl-CoA and other organic molecules that enter the TCA cycle. The metabolic flexibility afforded by multiple inputs into the TCA cycle allows cancer cells to adequately respond to the fuels available in the changing microenvironment during the evolution of the tumor (Boroughs and DeBerardinis 2015).

Furthermore, the catabolism of BCAAs also provides an important source for the generation of amino acids, especially glutamine and alanine. Different cancer studies (Lasagna-Reeves et al. 2010; Gao et al. 2008; Zira et al. 2010) have shown alterations in glutamine levels that are presumably associated with increased metabolic activity derived from the conditions of hypoxia and hypermetabolism observed in the tumor environment (Eigenbrodt et al. 1998). Proliferating cancer cells take up glutamine and convert it to glutamate through a variet of deamidation and transamidation reactions, most notably the mitochondrial amidohydrolase glutaminase (Hensley et al. 2013). It leads to the production of ammonia and glutamate to balance the pH in tumor cells and could explain the increase of glutamate observed in the urine of PCa patients. This result is also in agreement with previous PCa studies performed in serum (Giskeødegård et al. 2015) and prostate tissue (McDunn et al. 2013). Glutamate is subsequently transformed into α-ketoglutarate through a series of biochemical reactions termed glutaminolysis that contribute to replenish depleted intermediates of the TCA cycle (DeBerardinis et al. 2008).

Regarding amino acids metabolism, a significant decrease of 4-imidazole-acetate, a compound linked to histidine metabolism, was also observed in the urine of PCa patients. Interestingly, this metabolite was also identified in a previous study carrried out with serum samples collected up to 20 years prior to PCa diagnosis (Mondul et al. 2015). In this study, it was associated with both the overall risk of PCa (odds ratio 1.33) and aggressive PCa (odds ratio 1.40). Previous studies have also shown that histidine levels are increased in serum (Giskeødegård et al. 2015) and tissue (McDunn et al. 2013) samples from PCa patients, our finding perhaps reflecting a limited ability to process this amino acid by PCa cells. Alterations in histidine metabolism, as well as in BCAA (valine, leucine and isoleucine) metabolism, have also been observed in other cancers (e.g., ovarian cancer, breast cancer) (Ke et al. 2015; Schramm et al. 2010).

An increase in the urinary levels of pseudouridine, an isomer of the nucleoside uridine in which the uracil moiety is attached through a carbon–carbon bond, was found to be elevated in the urine metabolomic profile of PCa patients compared with individuals diagnosed with BPH. Increased levels of uracil, or other uracil-containing metabolites (e.g., 2′-deoxyuridine) (Mondul et al. 2015), have been found in previous PCa studies (Jiang et al. 2010; McDunn et al. 2013; Spur et al. 2013; Sreekumar et al. 2009) suggesting an important role of the metabolism of this compound in this disease. Alterations in the levels of pseudouridine have also been observed in other pathological processes (Rasmuson and Bjork 1995; Vicente-Munoz et al. 2015; Masaki et al. 2006) and have been associated with disease activity, tumor burden, and clinical status (Tamura et al. 1987).

Finally, the analysis of the urinary metabolomic profiles of PCa patients and individuals diagnosed with BPH also revealed significant variations in the levels of fumarate, a key molecule in the TCA cycle. Within this cycle, the succinate dehydrogenase (SDH) complex converts succinate to fumarate, that is further down transformed to malate by the fumarate hydratase (FH). Mutations in these enzymes have been previously linked to renal cell carcinomas, uterine and skin cancer (Tomlinson et al. 2002). Previous studies have also shown decreased levels of other TCA metabolites (isocitrate, aconitate and succinate) in the urine of PCa patients, all the data supporting a disruption in energy metabolism (Struck-Lewicka et al. 2015). Moreover, the decreased levels of fumarate in the urine of PCa patients, compared with individuals diagnosed with BPH, positively correlates with previous studies showing an accumulation of this metabolite in PCa bone metastases (Thapar and Titus 2014) and prostate tissue (McDunn et al. 2013), a process that would lead to a reduction in the levels of circulating fumarate. Interestingly, succinate, another metabolite exhibiting decreased levels in the urine of PCA patients, also tends to accumulate in cancer cells. Both metabolites belong to a family of compounds termed oncometabolites that are known to accumulate in cancer cells and facilitate cancer progression (Yang et al. 2013). In particular, these two oncometabolites have been associated with the aberrant stabilization of HIF-1a (Semenza 2010), a key protein in cancer that is commonly overexpressed in PCa cells (Thomas and Kim 2008).

## Concluding remarks

In summary, the present study reveals for the first time that the analysis of urinary metabolomic profiles provides a non-invasive tool for characterizing PCa-associated biomarkers and for getting a better understanding of the metabolic alterations underlying this neoplastic process. Although further validation of the results, using an independent set of samples, will be necessary to increase the robustness of this analysis, our data support the idea that multivariate statistical analysis of ^1^H-NMR urinary metabolomic profiles obtained from PCa patients could be used for objectively discriminating individuals with BPH or PCa.

## Abbreviations

PCa: Prostate cancer
PSA: Prostate specific antigen
DRE: Digital rectal examination
^1^H-NMR: Nuclear magnetic resonance
BPH: Benign prostatic hyperplasia
TSP: Trimethylsilylpropionic acid-d_4_ sodium salt
CPMG: Carr-Purcell-Meiboom-Gill
FIDs: Free induction decays
1D: One-dimensional
2D: Two-dimensional
PQN: Probabilistic quotient normalization
PCA: Principal component analysis
OPLS-DA: Orthogonal partial least squares discriminant analysis
VIP: Variable importance in projection
BMI: Body mass index
BCAA: Branched-chain amino acids
LC–MS: Liquid chromatography–mass spectrometry
GC–MS: Gas chromatography–mass spectrometry
GNMT: Glycine-*N*-methyltransferase
SARDH: Sarcosine dehydrogenase
DMGDH: Dimethylglycine dehydrogenase
TCA: Tricarboxylic acid
SDH: Succinate dehydrogenase
FH: Fumarate hydratase
